# Miao medicine Tiekuaizi (*Radix Chimonanthi*): a review of its traditional uses, phytochemistry, pharmacology, and predictive analysis on quality markers

**DOI:** 10.3389/fphar.2024.1491585

**Published:** 2025-01-03

**Authors:** Li-ying Luo, Xu-dong Zhang, Hui-min Mei, Jian-hui Ma, Xin Peng, Ming-hong Dong, Cong Huang

**Affiliations:** ^1^ Guizhou University of Traditional Chinese Medicine, Guiyang, China; ^2^ Anshun Hospital of Traditional Chinese Medicine, Anshun, China

**Keywords:** Miao medicine Tiekuaizi, Radix Chimonanthi, phytochemistry, pharmacology, quality marker

## Abstract

Miao medicine Tiekuaizi (*Radix Chimonanthi*) has been successfully used by Guizhou Miao ethnic physicians in clinical treatments, demonstrating significant curative effects. The research progress on the resource distribution, traditional uses, phytochemistry, and pharmacology of Tiekuaizi was reviewed by collecting related literature from traditional Chinese medicine books and various databases. Based on the comprehensive review and centered around the principles of traditional Chinese medicine quality marker (Q-Marker) theory, nine secondary metabolites (scopoletin, scopolin, isofraxidin, fraxin, scoparone, calycanthoside, 6, 7, 8-trimethoxycoumarin, tomenin, and calycanthine) are suggested as potential Q-markers for Tiekuaizi to establish quality control standards. This can provide a valuable reference for the collection and processing, pharmaceutical production, and effectiveness and safety of clinical applications of Miao medicine Tiekuaizi.

## Graphical Abstract

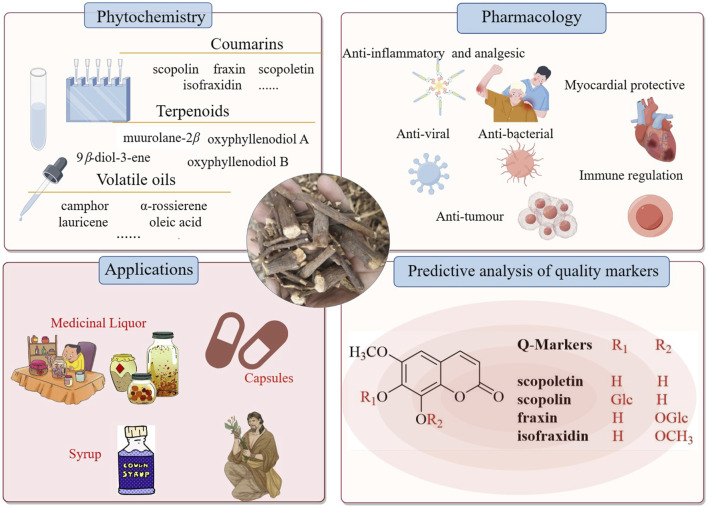

## 1 Introduction

Miao medicine Tiekuaizi (*Radix Chimonanthi*) is the dried fine root of *Chimonanthus praecox* (L.) Link and *Chimonanthus nitens* Oliv, a genus of Calycanthaceae, a characteristic ethnic medicine of Guizhou, with the effect of removing wind and alleviating pain (QuFengZhiTong) and circulating blood and removing toxins (HuoXueJieDu). It is used for treating asthma, strain and cough, stomach pain, abdominal pain, rheumatism, paralysis, boils and swellings, bruises, injuries, etc. It has been widely used in clinics and has also formed a large-scale pharmaceutical industry, such as JinGuShang spray, TongLuoGuZhiNing ointment, FuFangXianLingFengShi liquor, ShengLongQuFeng medicinal liquor, FuFangXieTeng medicinal liquor, MaLanGanHan capsules, Liangjiang Weiyang capsules, Ganqing syrup, and other special varieties (Guizhou Provincial Drug Administration, 2019). Tiekuaizi was first recorded in the *Materia Medica* and is also included in *the Chinese Materia Medica (Miao Medicine Volume)* (Qiu et al., 2006), *Zhong Yao DaCi Dian* (Nanjing University of Traditional Chinese Medicine, 2006), and the 2019 edition of *the Guizhou Quality Standards for Chinese and Ethnic Medicinal Herb* (Guizhou Provincial Drug Administration, 2019). However, the content only includes the plant source, morphology, and identification of medicinal materials. There are no records of the index ingredients of Miao medicine Tiekuaizi, and there is no standard basis for systematically explaining the relationship between “ingredients – quality – efficacy,” which does not meet the requirements of traceability, cultivation, preparation, processing, and quality control. Therefore, the construction of a quality evaluation system that correlates traditional efficacy and clinical efficacy is an important issue for the development of the Tiekuaizi industry and the safety of medicine.

Since the last century, domestic and foreign scholars have studied the genus *Chimonanthus* and found that the genus is rich in volatile oils, coumarins, and flavonoids (Li et al., 2018; Liu et al., 2018; Wang et al., 2012; Shu et al., 2019; Li et al., 2021; He et al., 2023; Lin, 2019). At present, there are many research reports on the properties of leaves of *C. nitens* and *C. praecox*, and many researchers have reviewed their metabolites and pharmacological activities (Ma et al., 2010; Nie et al., 2009; Dong et al., 2021; Yu et al., 2021; Hu and Yang, 2008). However, relatively few studies have been conducted on Tiekuaizi (*Radix Chimonanthi*, roots of *C. nitens* and *C. praecox*). Additionally, geographical differences have resulted in a homonym phenomenon with *Helleborus thibetanus*, a member of the Ranunculaceae family. Therefore, a review of its resource distribution, clinical medication, chemical composition, and pharmacological effects has guiding significance for correct clinical medication.

Literature search was conducted with the keywords “*Radix Chimonanthi*,” “*C. praecox*,” and “*C. nitens*.” from Web of Science (https://webofscience.clarivate.cn), PubMed (https://pubmed.ncbi.nlm.nih.gov), China National Knowledge Infrastructure (https://www.cnki.net), Wanfang Database (https://www.wanfangdata.com.cn), and Google Scholar (https://scholar.google.com). Manual reading was performed to eliminate duplicate literature works and irrelevant content in the database. Only the research literature works mentioning the roots of *C. praecox* and *C. nitens* as medicinal parts were selected. Based on classic Chinese medicine books and relevant literature collected from various databases mentioned above, all eligible studies were analyzed and summarized. A review was conducted on the resource distribution, usage, phytochemistry, and pharmacological effects of the Miao medicine Tiekuaizi. With the continuous research on Tiekuaizi, coumarins, alkaloids, terpenoids, volatile oils, and other secondary metabolites have been isolated from it (Luo et al., 2023). Modern pharmacological studies have found that it has anti-inflammatory effects, analgesic effects, used in treatment of cardiovascular and cerebrovascular diseases, improves disorders of glucolipid metabolism, regulates immunity, and has anti-tumor and other effects (Li and Zou, 2018; Liu et al., 2018; Wang et al., 2012; Shu et al., 2019; Dong et al., 2021; Yu et al., 2021; Hu and Yang, 2008). However, no research has been conducted to systematically explain the quality evaluation of Tiekuaizi from the perspective of a quality marker (Q-marker), which limits the establishment of an evaluation system for the scientific quality of Tiekuaizi. Based on the existing research results of Tiekuaizi and the Q-marker theory of Chinese medicine (Liu et al., 2016), this paper analyzes and predicts the Q-marker of Tiekuaizi in terms of metabolite specificity, effectiveness, measurability, quality transfer traceability, and pharmaceutical rule of TCM (“five principles”) to provide a scientific basis for the establishment of a better quality evaluation standard of Tiekuaizi of Miao medicine.

## 2 Resource distribution and ethnic clinical medication

Tiekuaizi is widely distributed in Guizhou, Yunnan, Sichuan, and other provinces, and it also known as Zuangufeng, Yanma Sangen, and Tiegangcha. It has a long history of medicinal use in Guizhou (Guizhou Provincial Drug Administration, 2019; Zhu et al., 2020), and *the Guiyang Folk Medicinal Herbs* records that its formulations can treat iron injuries, stomach pain, cold abdominal pain, coughs from strain, and blood pockets in the abdomen of women (Guiyang Health Bureau, 1959); according to *the Chinese Materia Medica (Miao Medicine Volume)*, it can treat phuang poisonous sores, strain cough, stomach pain, abdominal pain, rheumatism paralysis, healing sores, swelling and poison, bruises, and injuries. Prepared formulations include Liangjiang Weiyang capsules, FuFangXieTeng medicinal liquor, MaLanGanHan capsules, and Ganqing syrup (Qiu et al., 2006). *The Colorful Atlas of Chinese Miao Medicines* records that it can treat rheumatic pain, stomach pain, and asthma (Wang, 2002); the *Research and Development of Miao Medicine in Guizhou* records the treatment of bone fractures and rheumatic joint pain (Bao and Ran, 1999); *the Chinese Folklore Hundred Herbs and Prescriptions* records the treatment of lumbar muscle strain and cold abdominal pain by grouping prescriptions (Zhou, 1998); *Guizhou Folk Remedies Collection* records treatment for wind paralysis, rheumatic pain, acute rheumatic arthritis, osteophytes, and conventional and new injuries (Yang and Yang, 1978). Different habitats, climates, and land resources will affect the properties of Tiekuaizi. On the other hand, with the annual development of the Tiekuaizi industry and strong market demand, it is urgent to improve its quality evaluation standards and provide the necessary scientific research base for industrial upgrading.

## 3 Phytochemistry

**FIGURE 1:**
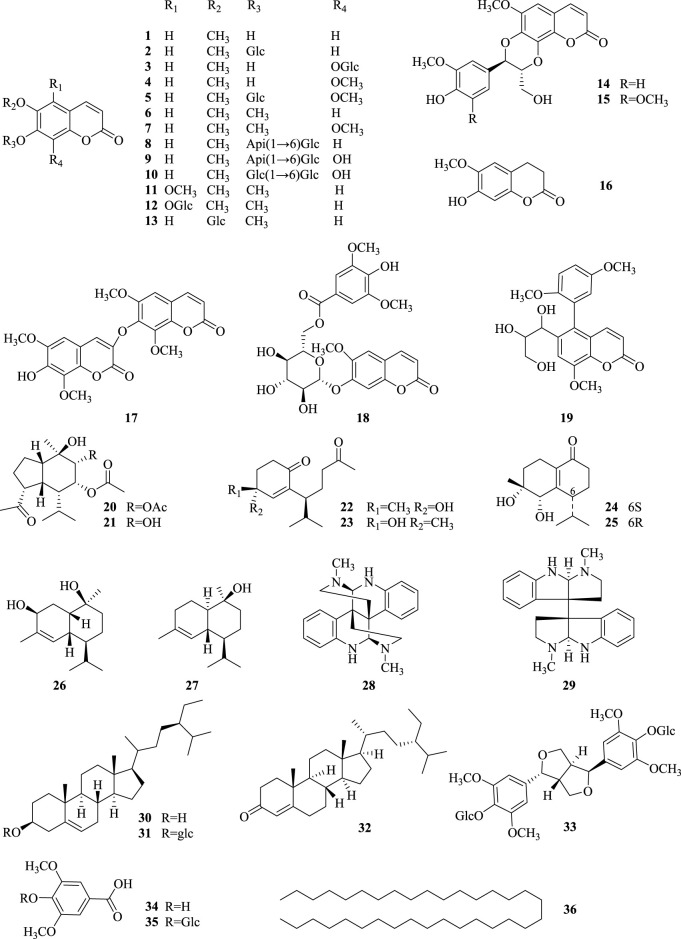
Structure of non-volatile metabolites of Tiekuaizi.

Since the last century, many researchers have been studying the secondary metabolites of the genus *Chimonanthus*. There have been many literature reports on the secondary metabolites from the leaves of *C. nitens and C. praecox*, but there have been relatively few reports on the research of roots (the medicinal part of Tiekuaizi). At present, more than 100 secondary metabolites have been isolated and identified from Tiekuaizi, including non-volatile compounds such as coumarins (**No. 1–19**), terpenoids (**no. 20–27**), and others (**no. 28–36**) and volatile oils (**no. 37–105**). Metabolite information can be found in Tables 1, 2. The structure of the non-volatile secondary metabolites obtained from Tiekuaizi is shown in Figure 1. Among them, coumarins, represented by scopoletin, are important pharmacodynamic markers of Tiekuaizi and have attracted attention due to their significant anti-inflammatory and analgesic effects.

**TABLE 1:** Non-volatile chemical composition of Tiekuaizi.

Category	No.	Metabolites	Formula	CAS	Source	Ref.
Coumarins	**1**	Scopoletin	C_10_H_8_O_4_	92-61-5	C	Li, D., (2008); Li, Q.J. et al. (2013); Tan, T. et al. (2017); Wang, X., (2020); Zhang, Y. (2013); Zhao, H.R. et al. (1993)
	**2**	Scopolin	C_16_H1_8_O_9_	531-44-2	C	Li, D., (2008); Li, Q.J. et al. (2013); Tan, T. et al. (2017); Wang, X. (2020); Zhao, H.R. et al. (1993)
	**3**	Fraxin	C_16_H_18_O_10_	524-30-1	C	Tan, T. et al. (2017); Wang, X. (2020); Zhang, X.D. et al. (2024)
	**4**	Isofraxidin	C_11_H_10_O_5_	486-21-5	C	Tan, T. et al. (2017); Wang, X. (2020); Zhang, Y. (2013); Zhang, X.D. et al. (2024)
	**5**	Calycanthoside	C_17_H_20_O_10_	483-91-0	C	Li, D. (2008); Li, Q.J. et al. (2013); Zhang, Y. (2013)
	**6**	Scoparone	C_11_H_10_O_4_	120-08-1	C	Li, D. (2008); Tan, T. et al. (2017); Wang, X. (2020); Zhang, Y. (2013)
	**7**	6, 7, 8-Trimethoxycoumarin	C_12_H_12_O_5_	6035-49-0	C	Li, D. (2008); Tan, T. et al. (2017); Zhang, Y. (2013)
	**8**	Xeroboside	C_21_H_26_O_13_	117,842-09-8	A	Li, Q.J. et al. (2013); Wang, X. (2020)
	**9**	Nitensoside A	C_21_H_26_O_14_	-	A	Li, Q.J. et al. (2013); Wang, X. (2020)
	**10**	Nitensoside B	C_23_H_30_O_15_	-	A	Li, Q.J. et al. (2013); Tan, T. et al. (2017)
	**13**	Magnolioside	C_16_H_18_O_9_	20,186-29-2	A	Tan, T. et al. (2017)
	**11**	5, 6, 7-Trimethoxycoumarin	C_12_H_12_O_5_	55,085-47-7	A	Li, Q.J. et al. (2013); Wang, X. (2020)
	**13**	Tomenin	C_17_H_20_O_10_	28,446-08-4	C	Li, D. (2008); Tan, T. et al. (2017)
	**14**	Clemiscosin A	C_20_H_18_O_8_	76,948-72-6	A	Tan, T. et al. (2017)
	**15**	Clemiscosin C	C_21_H_20_O_9_	84,575-10-0	A	Tan, T. et al. (2017)
	**16**	7-Hydroxy-6-methoxy-chroman-2-one	C_10_H_10_O_4_	90,843-91-7	B	Zhang, Y. (2013)
	**17**	Arteminorin A	C_22_H_18_O_10_	1,158,294-42-8	A	Tan, T. et al. (2017)
	**18**	7-O-(6-O-syringoyl-*β*-D-glucopyranosyl)-6-methoxycou-marin	C_25_H_26_O_13_	1,820,924-13-7	A	Wang, X. (2020)
	**19**	Praecoxin	C_21_H_22_O	1,698,917-27-9	B	Qian, H.B. (2016); Zhang, X.D. et al. (2024)
Terpenoids	**20**	Chimonol A	C_19_H_30_O_6_	1,574,775-65-7	B	Lou, H.Y. et al. (2018); Zhang, Y. (2013)
	**21**	Chimonol B	C_17_H_28_O_5_	1,574,775-69-1	B	Lou, H.Y. et al. (2018); Zhang, Y. (2013)
	**22**	Chimonol C	C_15_H_24_O_3_	1,569,669-10-4	B	Lou, H.Y. et al. (2018); Zhang, Y. (2013)
	**23**	Chimonol D	C_15_H_24_O_3_	1,569,669-08-8	B	Lou, H.Y. et al. (2018); Zhang, Y. (2013)
	**24**	Oxyphyllenodiol A	C_14_H_22_O_3_	363,610-30-4	B	Lou, H.Y. et al. (2018); Zhang, Y. (2013)
	**25**	Oxyphyllenodiol B	C_14_H_22_O_3_	363,610-32-6	B	Lou, H.Y. et al. (2018); Zhang, Y. (2013)
	**26**	Muurolane-2*β*, 9*β*-diol-3-ene	C_15_H_26_O_2_	23,971-80-4	B	Lou, H.Y. et al. (2018); Zhang, Y. (2013)
	**27**	(+)-t-cadinol	C_15_H_26_O	58,580-31-7	B	Lou, H.Y. et al. (2018); Zhang, Y. (2013)
Others	**28**	(+) - calycanthine	C_22_H_26_N_4_	595-05-1	C	Li, D., (2008); Li, Q.J. et al. (2013); Wang, X. (2020); Zhao, H.R. et al. (1993)
	**29**	Chimonanthine	C_22_H_26_N_4_	5545-89-1	B	Zhao, H.R. et al. (1993)
	**30**	*β*-Sitosterol	C_29_H_50_O	83-46-5	C	Li, D., (2008); Zhang, Y. (2013); Wang, X. (2020); Zhao, H.R. et al. (1993)
	**31**	Daucosterol	C_35_H_60_O_6_	474-58-8	B	Li, D., (2008); Zhao, H.R. et al. (1993)
	**32**	Sitostenone	C_29_H_48_O	1058-61-3	B	Zhang, Y. (2013)
	**33**	Liriodendrin	C_34_H_46_O_18_	573-44-4	A	Wang, X. (2020)
	**34**	Syringic acid	C_9_H_10_O_5_	530-57-4	B	Zhang, Y. (2013)
	**35**	Glucosyringic acid	C_15_H_20_O_10_	33,228-65-8	A	Wang, X. (2020)
	**36**	Tetratriacontane	C_34_H_70_	14,167-59-0	B	Li, D. (2008)

A is the roots of *C. nitens*; B is the roots of *C. praecox*; C is the roots of *C. nitens* and *C. praecox*

**TABLE 2:** Composition of volatile oils in Tiekuaizi.

*C. nitens*			*C. praecox*		
No.	Metabolites	Molecular formula	No.	Metabolites	Molecular formula
No.	Metabolites	Molecular formula	No.	Metabolites	Molecular formula
**37**	*α*-Thujene	C_10_H_16_	**71**	Camphene	C_10_H_16_
**38**	*α*-Pinene	C_10_H_16_	**72**	1, 8-Cineole	C_10_H_18_O
**39**	Camphene	C_10_H_16_	**73**	L-Linalool	C_10_H_18_O
**40**	Sabinene	C10H16	**74**	(−)-Borneol	C_10_H_18_O
**41**	*β*-Pinene	C10H16	**75**	Endobornylacetate	C_12_H_20_O_2_
**42**	*β*-Myrcene	C_10_H_16_	**76**	*α*-Copaene	C_15_H_24_
**43**	*α*-Terpinene	C_10_H_16_	**77**	Calamenene	C_15_H_22_
**44**	4-Isopropyltoluene	C_10_H_14_O	**78**	Seychellene	C_15_H_24_
**45**	Cineole	C_10_H_18_O	**79**	*α*-Humulene	C_15_H_24_
**46**	*γ*-Terpinene	C_10_H_16_	**80**	Aromadendrene	C_15_H_24_
**47**	Camphor	C_10_H_16_O	**81**	Germacrnen B	C_15_H_24_
**48**	Borneol	C_10_H_18_O	**82**	*α*-Muurolene	C_15_H_24_
**49**	Terpinine-4-ol	C_10_H_18_O	**83**	*γ*-Cadinene	C_15_H_24_
**50**	Linalyl propionate	C_13_H_22_O_2_	**84**	*δ*-Cadinene	C_15_H_24_
**51**	L-Menthol	C_10_H_18_O	**85**	*α*-Calacorene	C_15_H_20_
**52**	*β*-Citronellol	C_10_H_20_O	**86**	Nerolidol	C_15_H_20_
**53**	Geraniol	C_10_H_18_O	**87**	Caryophyllene oxide	C_15_H_24_O
**54**	Bornyl acetate	C_12_H_20_O_2_	**88**	*α*-Cubebene	C_15_H_24_
**55**	*α*-Copaene	C_15_H_24_	**89**	*α*-Cadinol	C_15_H_26_O
**56**	Geranylacetate	C_12_H_20_O_2_	**90**	T-muurolol	C_15_H_26_O
**57**	*β*-Caryophyllen	C_15_H_2_	**91**	Patchouli alcohol	C_15_H_26_O
**58**	Seychellene	C_15_H_24_	**92**	Myristic acid	C_14_H_28_O_2_
**59**	*β*-Patchoulene	C_15_H_24_	**93**	Pentadecanoic acid	C_15_H_30_O_2_
**60**	*α*-Amorphene	C_15_H_24_	**94**	Palmitoleic acid	C_16_H_30_O_2_
**61**	Germacrene	C_15_H_24_	**95**	Palmitic acid	C_16_H_30_O_2_
**62**	Naphthalene	C_15_H_24_	**96**	Linoleic acid	C_16_H_32_O_2_
**63**	*δ*-Cadinene	C_15_H_24_	**97**	Stearic acid	C_18_H_36_O_2_
**64**	*α*-Calacorene	C_15_H_20_	**98**	Hexanal	C_6_H_12_O
**66**	Xanthophyll	C_15_H_26_O	**99**	2-Heptanone	C_6_H_12_O
**67**	Azulene	C_15_H_24_	**100**	Heptanal	C_7_H_14_O
**68**	Valencene	C_14_H_18_O	**101**	*p*-Cymene	C_10_H_14_
**69**	Palmitic acid	C_16_H_32_O_2_	**102**	*dl*-Limonene	C_10_H_16_
**70**	Oleic acid	C_18_H_34_O_2_	**103**	Methyl salicylate	C_8_H_8_O_3_
			**104**	Myrtenol	C_10_H_16_O
			**105**	Myrtenal	C_10_H_14_O
			**42**	Myrcene	C_10_H_16_
			**47**	Camphor	C_10_H_16_O
			**60**	*α*-Amorphene	C_15_H_24_
			**70**	Oleic acid	C_18_H_34_O_2_

### 3.1 Non-volatile secondary metabolites

#### 3.1.1 Coumarins

Coumarins are widely found in the roots, stems, leaves, flowers, fruits, and seeds of higher plants and in animals and microorganisms. It is an important natural medicine and an important secondary metabolite for the study of Chinese and ethnic medicines. Coumarins often have hydroxyl and alkoxy substitutions on the parent nucleus, and the main metabolic modes include sulfonation, reduction reactions, and acidification of glucuronides (Kuang, 2023). Modern pharmacology has found that coumarins have a wide range of biological activities. Miao medicine Tiekuaizi mainly contains coumarins, which have anti-inflammatory, analgesic, and antibacterial effects (Zhao et al., 1993; Li, 2008; Zhang, 2013; Qian, 2016). Many coumarins obtained from the roots of *C. praecox* have been isolated and identified, including scopoletin, scopolin, 6,8-dimethoxy-7-hydroxycoumarin, 6,7,8-trimethoxycoumarin, 6,7-dimethoxycoumarin, tomenin, isozinpyridine, isozinpyridine 7-O-*β*-D-glucoside, 7-hydroxy-6-methoxy-chroman-2-one, and praecoxin, of which praecoxin is a new coumarin isolated by our research group (Qian, 2016).

In recent years, researchers have also studied the secondary metabolites of the roots of *C. nitens*. Li et al. (2013) isolated two new coumarins, namely, nitensosides A–B, and five known coumarins from them and determined their antibacterial activity.

Tan et al. (2017) first developed a method based on a modified mass defect filter (MDF) and performed a comprehensive analysis of coumarins from different medicinal parts of *C. nitens* by ultra-high liquid chromatography–tandem quadrupole time-of-flight mass spectrometry (UPLC-QTOF/MS). The MDF-based five-point screening method and the visual isotope ion technique allow rapid screening of precursor ions of interest. The mass spectrometry fragmentation patterns of coumarins were systematically studied, and 32 coumarins, including 19 potential new coumarins, were initially identified unequivocally in *C. nitens* roots. In 2020, Wang (2020) isolated 10 coumarins from the roots of *C. nitens* and compared the changes in the contents of four metabolites, namely, scopolin, fraxin, scopoletin, and isofraxidin, after drying in the shade, stoving, steaming, and carbonization.

Comparative analysis shows that the abundance of coumarins in the roots of *C. praecox* and *C. nitens,* among which there are eight co-contained coumarins, including scopoletin, scopolin, isofraxidin, fraxin, scoparone, calycanthoside, 6, 7, 8-trimethoxycoumarin, and tomenin.

#### 3.1.2 Terpenoids

In 2018, Lou et al. (2018) isolated four new (Chimonol A–D) and four known sesquiterpenoids (oxyphyllenodiol A, oxyphyllenodiol B, muurolane-2*β*, and 9*β*-diol-3-ene and (+)-t-cadinol) from the roots of *C. nitens*. The antimicrobial activity was evaluated by determining the minimum inhibitory concentrations (MICs) using the micro-broth dilution method.

#### 3.1.3 Others

Except for the above coumarins and terpenoids, Tiekuaizi also contains alkaloids, steroids, and phenolic acids (Zhao et al., 1993; Li, 2008; Wang, 2020), such as (+)-calycanthine, chimonanthine, *β*-sitosterol, daucosterol, sitostenone, liriodendrin, syringic acid, glucosyringic acid, and tetratriacontane. Some flavonoids are also present in Tiekuaizi, and Duan et al. (2013) studied the mechanism by which the total flavonoids of Tiekuaizi delay *D*-galactose-induced aging, possibly in relation to scavenging free radicals in the body and improving immune function. However, the isolation and identification of the flavonoid metabolites in Tiekuaizi have not been reported.

### 3.2 Volatile oils

Volatile oils are a mixture with complex secondary metabolites, ranging from tens to hundreds. The components of volatile oils are most commonly terpenoids, together with small non-terpenoid aliphatic and aromatic compounds (Kuang, 2023). Tiekuaizi has a pungent and aromatic flavor. The volatile oils in Tiekuaizi are mainly found in the phloem. Wang et al. (2010) identified 36 secondary metabolites from the volatile oil of the roots of *C. nitens* using GC–MS, with higher contents of cineole (25.29%), terpinine-4-ol (13.07%), linalyl propionate (10.50%), camphor (7.27%), oleic acid (6.98%), borneol (4.17%), and sabinene (4.10%).

Gao et al. (2011) and Lu et al. (2021) successively analyzed the components of the volatile oil from the roots of *C. praecox* using the GC–MS method, and 30 and 33 secondary metabolites were detected, respectively, with the main metabolites being the same, including palmitic acid, linoleic acid, oleic acid, and *α*-cadinol. In contrast, Lu et al. (2021) did not detect the following eight metabolites: aromadendrene, *α*-amorphene, germacrnen B, *α*-muurolene, *α*-cubebene, patchouli alcohol, myristic acid, and pentadecanoic acid) and detected the following ten more metabolites containing hexanal, 2-heptanone, camphor, myrcene, *p*-cymene, *dl*-limonene, methyl salicylate, myrtenol, and myrtenal.

Comparative analysis of the volatile oil metabolites in *C. praecox* and *C. nitens* showed significant differences, with only camphor, lauricene, *α*-rossierene, and oleic acid being the co-contained volatile oils, as shown in Table 2.

In summary, Tiekuaizi is rich in volatile oils, coumarins, alkaloids, and terpenoids. Tiekuaizi is a multi-resource Chinese medicine from two species, *C*. *praecox* and *C. nitens*, in the *Chimonanthus* genus, and the secondary metabolites of Tiekuaizi have not been fully studied from the number of compounds. Based on the existing research of Tiekuaizi, there are only 14 co-contained components, including 10 non-volatile secondary metabolites (**No. 1–7, No. 13, No. 28,** and **No. 30**) and four volatile oils (**No. 42, No. 47, No. 60,** and **No. 70**). The roots of *C. praecox* and *C. nitens* are used as Tiekuaizi for clinical medicine. The quality control of co-contained metabolites and the correlation between co-contained metabolites and drug efficacy need to be studied. In addition, according to the research reports on the secondary metabolites of the other medicinal parts of *C. nitens* and *C. praecox* (stems, leaves, flowers, seeds, etc.) (Li et al., 2021), it can be observed that the roots are very different from the other parts. In the future, a modern high-throughput component identification technology can be used to explore the commonality and differences of secondary metabolites in different medicinal parts of the two plants and combined with pharmacological research to study the therapeutic material basis of Tiekuaizi.

## 4 Pharmacological effects

Modern pharmacology has shown that Miao medicine Tiekuaizi has pharmacological effects including anti-inflammatory, analgesic, immune-regulating, and anti-tumor effects; treating cardiovascular and cerebrovascular diseases; and improving disorders of glycolipid metabolism (Table 3). The pharmacologically active metabolites are coumarins, volatile oils, and so on. The main active monomers are the coumarin compounds such as scopoletin, scopolin, isofraxidin, and fraxin.

**TABLE 3:** Pharmacological effects of extract or main components in Tiekuaizi.

Activity	Sample	Model/Assay	Dosage	Positive control drug	Effect/mechanism	Ref.
Anti-aging	Total flavonoids	*D*-galactose induced aging in mice	High: 5 g/kg/d Low: 2.5 g/kg/d (continuous administration for 3 weeks)	Vit E: 0.1 g/k/d	Total flavonoids of Tiekuaizi can increase the index of thymus, spleen, and brain in mice. It is suggested that enhancing immunity may be one of the ways to delay aging of total flavonoids of Tiekuaizi.	Duan et al. (2013)
Anti-cancer	Volatile oil	Human gastric cancer cells BGC-823	300, 150, 75, 35, and 15 μL/mL (the drug acts on the cells for 24 h)	Capecitabine: 0, 0.25, 0.5, 1.0,2.0, and 4.0 μL/mL	The volatile oil of *Chimonanthi Radix* could inhibit the growth of BGC-823 cells. Human gastric cancer cells BGC-823:IC_50_ = 11.793 μL/mL.	Lu et al. (2021)
	Isofraxidin	Breast cancer stem cells	0, 17, 50, 150, and 450 μmol/L	N/A	Isofraxidin can downregulate the expression of the Bcl-2 gene and activate the caspase gene. Family can induce apoptosis of breast cancer stem cells and inhibit their proliferation and migration at the same time.	Li et al. (2018)
Anti-inflammatory	Alcohol extract	Adjuvant arthritis rats	High: 7.0 g/kg/d Middle: 3.5 g/kg/d Low: 1.75 g/kg/d (the drug was administered once daily for 30 days)	Tripterygium wilfordii glycoside tablets: 31.5 mg/kg/d	The alcohol extract of Tiekuaizi can inhibit the foot swelling of AA rats and reduce the concentration of TNF-α and PAF in serum.	Li et al. (2017)
					Alcohol extract of Tiekuaizi downregulates the expressions of HIF-1 α, VEGF, and MMP-3.	Li and Qian (2016)
	Volatile oil		High: 10.0 g/kg Middle: 5.0 g/kg Low: 2.5 g/kg (continuous administration for 21 days)	Fengshi Gutong Capsule suspension: 0.2 g/kg	The volatile oil of Tiekuaizi can significantly inhibit the contents of IL-1β and TNF-α in serum of AA rats, reduce foot swelling and eliminate inflammation.	Qian et al. (2012b)
		Mouse ear swelling model	High: 20 g/kg Middle: 10 g/kg Low: 5 g/kg (continuous administration for 21 days)	Aspirin: 0.2 g/kg	The volatile oil of Tiekuaizi has good anti-inflammatory effects on acute, early and late inflammation.	Qian et al. (2012c)
	Aqueous extract	Osteoarthritis rats	High: 150 mg/kg Middle: 50 mg/kg Low: 16.5 mg/kg	Celecoxib: 16.7 mg/kg	Tiekuaizi significantly affects the NLRP3 inflammasome, which is regulated by the HIF-1α pathway. Tiekuaizi inhibited pyroptosis and reduced synovial inflammation.	Zhang et al. (2024)
		Collagen-induced arthritis rats	High: 1.0 g/kg/d Middle: 0.5 g/kg/d Low: 0.25 g/kg/d (three times a week, Continuous gavage treatment for 25 days)	Methotrexate: 2.0 mL/kg/d	Tiekuaizi may alleviate the inflammatory response in RA rats through an anti-rheumatoid arthritis mechanism involving the OPG/RANK/RANKL signaling pathway.	Shan et al. (2023)
	Fraxin	(Mouse mononuclear macrophage cell line) RAW 264.7 cells	20 μg mL^-1^(the drug acts on the cells for 24 h)	N/A	Fraxin can have anti-inflammatory effects by clearing intracellular reactive oxygen species (ROS), reducing intracellular oxidative stress, inhibiting macrophages to promote M1 phenotype polarization, and promoting anti-inflammatory M2 phenotype polarization.	Wei et al. (2022)
	Isofraxidin	Osteoarthritis rats	*In vitro* study: 20 μM 10 μM, and 5 μM *In vivo* assessment: 20 mg/kg/day	N/A	Isofraxidin inhibits TLR4/of human OA chondrocytes. Activation of MD-2 axis and NF-κ B signal can promote cell proliferation and inhibit LPS induction, leading to inflammation.	Jin et al. (2018)
Anti-inflammatory and anti-oxidant	Fraxin	Osteoarthritis chondrocyte induced by LPS	0, 0.3125, 0.625, 1.25, 2.5, 5, 10, 20, 40, 80, and 160 μg/m L (The drug acts on the cells for 24 h)	N/A	Fraxin can inhibit the overexpression of inflammatory factors and catabolic genes, promote the upregulation of cartilage-specific genes, eliminate ROS, and has good anti-inflammatory, antioxidant and protective effects on chondrocytes	Zhan et al. (2021)
Anti-inflammation and analgesia	Compound Q-1	Human rheumatoid arthritis fibroblast synovial cell line (MH7A)	High: 100 mg/L Middle: 50 mg/L Low: 25 mg/L	Methotrexate: 5 μmol/L	The alcohol extract of Tiekuaizi has obvious analgesic effect, and its action site may be the peripheral nerve endings or the central nervous system. Compound Q-1 may inhibit the proliferation and migration of MH7A cells and reduce the secretion of inflammatory cytokines TNF-α and IL-6 by regulating p65 and I κ B α-mediated NF-κ B signal pathway and p38-mediated MAPK signal pathway.	Wu et al. (2021)
Analgesia	Volatile oil	Kunming mice	High: 20 g/kg Middle: 10 g/kg Low: 5 g/kg (the drug was given intragastric once a day for 7 days)	Aspirin: 0.2 g/kg	The LD_50_ of volatile oil of Tiekuaizi 8.50 mL kg^-1^. Tiekuaizi volatile oil can obviously inhibit the writhing reaction of mice and increase the pain threshold of thermal stimulation in mice.	Qian et al. (2012d)
	Alcohol extract	Mouse model of pain	High: 200 mg/kg Middle: 100 mg/kg Low: 50 mg/kg	Tramadol hydrochloride: 15 mg/kg	The alcohol extract of Tiekuaizi has inhibitory effect on a variety of pain models, showing obvious analgesic effect, and has a certain dose and time dependence.	Qian et al. (2010)
Protects the myocardium	Isofraxidin	Rat model of acute myocardial infarction after atherosclerosis	High: 93.1 mg/mL Middle: 46.585 mg/mL Low: 23.293 mg/mL (the drug was administered intragastric once a day for 28 days)	Fufang Danshen dropping pills: 13.5 mg/mL	Isofraxidin by suppressing NLRP3 inflammatory bodies plays a cardioprotective role in myocardial infarction.	Xu et al. (2022)
	Alcohol extract	Myocardial ischemic rat model	High: 465.5 mg/kg Middle: 232.925 mg/kg Low: 119.615 mg/kg (The drug was injected intraperitoneally for 7 days)	Fufang Danshen dropping pills: 67.5 mg/kg	The alcohol extract of Tiekuaizi can reduce the level of serum TNF-α, inhibit the expression of inflammatory factors, play an anti-inflammatory effect, and have a certain protective effect on myocardial ischemia. The indexes of CK-MM, and CK-MB in high, middle and low dose groups of alcohol extract of Tiekuaizi were lower than those in model group.	Li et al. (2019)
Anti-tumor and improves body immunity	Isofraxidin	Liver cancer patient	High: 0.35 mg/kg Middle: 0.25 mg/kg Low: 0.15 mg/kg (Once a day, twice a week)	Chemotherapy, surgery, and other treatments	Isofraxidin can effectively inhibit the growth of MMP-7 and cancer cells in patients with liver cancer and reduce the invasive ability of cancer cells.	Chen et al. (2016)
Enhances immunity	Water decoction	Immunocompromised mouse model	High: 1.00 g/mL Middle: 0.50 g/mL Low: 0.25 g/mL (the drug was administered intragastric once a day for 30 days)	Astragalus granule: 0.50 g/mL	Tiekuaizi can enhance immunity, increase thymus index, macrophage phagocytosis, regulate the levels of cytokines, IgM, complement C3 and C4, and the number of RBC and WBC to enhance immune ability and improve immunosuppression.	Ding et al. (2021)
Lipid-lowering	Scopoletin	Obese mouse model	High: 20 mg/kg Low: 10 mg/kg (The drug was given intragastric once a day for 4 weeks)	Silymarin: 30 mg/kg	Scopoletin can improve the blood lipids and fat accumulation of obese mice induced by HFD- and significantly reduce the cell size of adipose tissue of accessory testis in mice.	Jin et al. (2021)

### 4.1 Anti-inflammatory and analgesic effects

The alcohol extract of Tiekuaizi can significantly reduce the levels of hypoxia-inducible factor 1-subunit alpha (HIF-1α) and tumor necrosis factor-α (TNF-α) in the serum, as well as the expression of joint matrix metallo proteinase-3 (MMP-3) and vascular endothelial growth factor (VEGF) related to joint swelling and destruction, in the treatment of arthritis in rats (Li et al., 2017; Li and Qian, 2016; Shan et al., 2023; Zhang et al., 2024). In the treatment of rats, a model of myocardial ischemia, the alcoholic extract of Tiekuaizi reduced the expressions of interleukin-18 (IL-18), interleukin-6 (IL-6), and TNF-α and was able to protect against myocardial cell injury produced by inflammatory cell infiltration (Li et al., 2019). The volatile oil of Tiekuaizi significantly inhibited ear swelling in a model of xylene-induced auricular swelling in mice, and it also inhibited the swelling and granulation of the foot and plantar area in mice caused by carrageenan, showing definite anti-inflammatory effects (Qian et al., 2012b; Qian et al., 2012c).

The extracts from various parts of Tiekuaizi have analgesic and anti-inflammatory effects (Zhang, 2013). The analgesic effect was best in the high-dose group at the ethyl acetate site, followed by the low-dose group at the petroleum ether site; the anti-inflammatory effect was best in the low-dose group at the water site, followed by the high-dose group at the ethyl acetate site. In terms of analgesia, the Tiekuaizi alcoholic extract and volatile oil reduces the acetic acid-induced writhing response in mice and significantly increases the pain threshold in the heat radiation tail-flick test and hot plate method in mice (Qian et al., 2010; Qian et al., 2012d; Qian et al., 2012a).

Our research team discovered coumarin monomer compound Q-1 (praecoxin) in the previous study on Tiekuaizi, which can inhibit MAPK and NF-κB signaling pathways and reduce the expressions of inflammatory factors such as IL-6 and TNF-*α*, and it can also inhibit the cell proliferation of human rheumatoid arthritis (RA) fibroblast-like synovial cell lines, exerting an anti-rheumatoid arthritis effect (Wu et al., 2021).

Tiekuaizi has good anti-inflammatory and analgesic effects as it is rich in coumarins. Studies have shown that coumarin metabolites, such as scopoletin, isofraxidin, and fraxin, can mostly inhibit histamine, NF-κB, and MAPK pathways; inhibit the release of cellular inflammatory factors such as IL-1*β*, IL-6, and TNF-*α*; and avoid excessive activation of the immune system, resulting in powerful anti-inflammatory effects (Wei et al., 2009).

### 4.2 Myocardial protective effect

The alcohol extracts of Tiekuaizi can reduce triglyceride and low-density lipoprotein levels and increase high-density lipoprotein levels in a model of myocardial ischemia in rats, achieving lipid-lowering effects. It also reduces serum creatine kinase (CK) CK-MB and CK-MM levels; decreases the expression of TNF-α, IL-6, and IL-18 during myocardial ischemia; and reduces myocardial damage caused by ischemia (Li et al., 2019).

The Tiekuaizi alcohol extract can downregulate IκB-α, NF-κBp65, and NF-κBp50 mRNA and protein expressions, inhibit the activation of the NF-κB signaling pathway, reduce the inflammatory response, and play a cardioprotective role, which has a good protective effect on the cardiac function of rats in a myocardial ischemic model (Xu et al., 2022). Studies have shown that the coumarin analog isofraxidin alleviates myocardial infarction by inhibiting NLRP3 inflammasome activity (Chen et al., 2020).

### 4.3 Anti-viral and anti-bacterial effects

The volatile oil of the roots of *C. praecox* has the strongest antibacterial activity, and the compositional analysis showed mainly more monoterpenes than in the leaves and peel. It is possible that this metabolite is the active metabolite of the root volatile oil in inhibiting fungi, especially against pathogenic fungi and Gram-positive bacteria, with a broader spectrum of inhibitory activities (Wang et al., 2009). It is, therefore, customary in folklore to use Tiekuaizi for inflammatory conditions of the gastrointestinal tract, presumably in relation to its strong antibacterial properties.

In an *in vitro* test, the metabolite calycanthine found in Tiekuaizi showed significant inhibitory activity against five phytopathogenic fungi *Exserohilum turcicum, Bipolaris maydis, Alternaria solani, Sclerotinia sclerotiorum, and Fusarium oxysporum*, with *B. maydis* being the most sensitive with an EC50 value of 29.3 mg mL^-1^ (Zhang et al., 2009).

The metabolites chimonol C, chimonol D, oxyphyllenodiol A, oxyphyllenodiol B, muurolane-2β, and 9β-diol-3-ene, isolated from *C. praecox* roots, showed inhibitory activity against *Staphylococcus smoothus* (ATCC 2001) and *Staphylococcus aureus* (ATCC 43300) with MIC values of 128–197 μg mL^-1^. The metabolite chimonol A–D showed antibacterial activity against *S. aureus* (ATCC 25923) with MIC values of 162–254 μg mL^-1^ (Lou et al., 2018).

### 4.4 Anti-tumor effects

In cell proliferation inhibition experiments, it was found that Tiekuaizi could significantly inhibit the growth and proliferation of BGC-823 tumor cells *in vitro*, and the inhibitory effect was positively correlated with the concentration (Lu et al., 2021). It has been reported that isofraxidin can synergistically produce anti-tumor effects by blocking the tumor cell cycle in the G2/M phase, inducing apoptosis, and reducing tumor cell invasion (Chen et al., 2016; Li et al., 2018).

### 4.5 Immune regulation

When immunity is disturbed, it can cause severe hypersensitivity reactions and diseases such as RA and immunodeficiency. Tiekuaizi, on the other hand, has a good immunoprotective effect and can enhance the immune capacity of immunosuppressed mice, increase the thymic index and the body weight of immunosuppressed mice, and enhance hypersensitivity reactions by a mechanism related to the increased secretion of expression factors such as IL-6, TNF-α, IgM, and complement C3; on the other hand, Tiekuaizi can also act on blood cells and increase the number of peripheral blood leukocytes, while the phagocytic index of phagocytes decreases (Ding et al., 2021).

Research has found that scopoletin, fraxin, and isofraxidin can avoid over-activation of the immune system by inhibiting macrophage polarization, inhibiting NF-κB and MAPK pathways, and suppressing the release of IL-1β, IL-6, and TNF-α cellular inflammatory factors (Chu et al., 2019; Wei et al., 2022; Jin et al., 2018). Tiekuaizi has strong regulatory effects on the immune system and contains more than three metabolites. The above research results may provide a theoretical basis for the use of Tiekuaizi in the treatment of autoimmune diseases.

### 4.6 Anti-aging effects

Tiekuaizi also has anti-aging effects, which can enhance human cognitive and learning abilities. The flavonoids in Tiekuaizi have a delaying effect on d-galactose-induced aging in experimental animals. The anti-aging mechanism of Tiekuaizi is probably through increasing the content of SOD, which enhances the body’s anti-aging system and increases the body’s ability to remove peroxidized lipid products, thus achieving anti-aging effects. It also achieves the anti-aging effect in experimental animals by reducing the number of free radicals in the body and enhancing the immunity in the body and by reducing the number of free radicals in the body and alleviating the neuroimmune damage to the central nervous system (Duan et al., 2013).

Tiekuaizi volatile oil improved the learning and memory capacity in CCH-induced VCI rats, improved the neuronal cell structure, increased the number of Nisus vesicles in the brain, inhibited apoptosis, upregulated serum BDNF levels and hippocampal ChAT activity, decreased hippocampal AChE activity, activated the BDNF/TrkB/PI3KJAkt signaling pathway, improved exploratory capacity, and increased the liking for novelty in rats (Ding et al., 2022).

### 4.7 Others

Research studies have shown that scopoletin can significantly reduce the expression of cholesterol-regulatory element-binding proteins 1 alpha (SREBP-1*α*), peroxisome proliferator-activated receptor *γ* (PPAR-*γ*), and other genes in mouse epithelial fat. The expression of genes such as proliferator-activated receptor *γ* (PPAR-*γ*) (Jin et al., 2021) reduces lipid synthesis, inhibits α-glucosidase and α-amylase activities to lower postprandial glucose, and also inhibits protein tyrosine phosphatase 1B (PTP1B) activity. It also inhibits the expression of protein tyrosine phosphatase 1B (PTP1B) and reduces insulin resistance (Jang et al., 2018), regulating glucolipid metabolism in several ways. Based on the methods and techniques of network pharmacology, it was found that Tiekuaizi mainly treats cerebral ischemic stroke (CIS) through key targets such as NOS3, SRC, and PPARG based on neuroactive ligand–receptor interactions and calcium signaling pathways by promoting revascularization, neuroprotection, and anti-inflammation, which also reflects the characteristics of Chinese medicine such as multi-target and multi-linkage in the treatment of CIS (Wang et al., 2022). In addition, volatile oil is a natural transdermal absorption enhancer that promotes drug absorption, is less irritating to the skin, and can play a certain therapeutic role. It has been found that the drug is more effective when administered transdermally, and volatile oil from Tiekuaizi can be added to microemulsions as a transdermal absorption enhancer, and volatile oils can be combined with microemulsions for better efficacy (Lu et al., 2020).

In summary, Tiekuaizi contains a variety of medicinal metabolites that synergistically induce anti-inflammatory, analgesic, antibacterial and antiviral, cardioprotective, immunomodulatory, and anti-tumor effects through a variety of pathways and has great clinical use. These effects are closely related to its abundance of coumarins, alkaloids, and terpenoids as active components. However, Tiekuaizi is rich in various substances, and current research is not comprehensive. Further in-depth research is needed on the pharmacological substance basis.

## 5 Toxicity

According to the Guizhou Provincial Drug Administration, quality standards for Chinese herbal medicines and ethnic medicinal materials in Guizhou Province, the Miao medicine Tiekuaizi has slight toxicity. There are reports (Knapp et al., 2023) that acute tonic–clonic seizures, tinnitus, disappearance of pupil light reflex, and abnormal vital signs occurred when sheep consumed the leaves of *C. praecox* in excess, and it is presumed that the toxicity of Tiekuaizi is related to the large amount of calycanthine detected in the stomach contents. Alkaloids are a class of natural organic compounds containing nitrogen, widely existing in plants, and most of them have strong physiological activities. Poisoning or death can occur when the dose is incorrectly administered (Zhu and Lu, 1992). Toxicity studies on Tiekuaizi showed that the volatile oil of Tiekuaizi could make mice curl up and sleep for approximately 20 min; mild toxicity cases showed abdominal convulsions and severe toxicity cases showed respiratory depression and even death, and the mice’s heart, liver, kidney, and other important organs showed no significant changes, with an LD_50_ value of 8.50 mL/kg. The analysis indicated that the volatile oil of Tiekuaizi has lower toxicity, being equivalent to 850 times the daily clinical dose (15 g) for an adult weighing 60 kg (Qian et al., 2012d). Acute toxicity was detected by the concentration gradient of the alcohol extract (0.25 g/kg, 0.50 g/kg, 1.00 g/kg, 2.00 g/kg, 4.00 g/kg, 8.00 g/kg, and 16.00 g/kg). The results showed that the concentration above 1.00 g/kg could cause death in rats, so 1.00 g/kg was considered the maximum safe dose (Shan et al., 2023). The LD_50_ values of charcoal products dried in the shade and subjected to stoving, steaming, and carbonization were 4,118.13 mg/kg, 3,733.36 mg/kg, 1,643.61 mg/kg, and >10,000 mg/kg, respectively. The toxic reactions were hind limb extension, stiffness and shivering, etc. The target organs may be the central nervous system and the neuromuscular system. By comparing the contents and toxicity of four coumarins across four Tiekuaizi products, it was found that while there was no significant difference in the content of two glucoside toxic metabolites, the levels of these metabolites were significantly reduced, and the carbonized charcoal products were non-toxic (Wang et al., 2020). Although there is evidence that the toxicity of Tiekuaizi may be related to its volatile oils, coumarins, and alkaloids, these secondary metabolites are also the main active components in Tiekuaizi. Consequently, it remains unclear whether the toxic components of Tiekuaizi are also its pharmacodynamic components. Furthermore, the mechanisms underlying the toxic reactions associated with Tiekuaizi and the detoxification processes following carbonization are also not well understood. For example, what happens to the chemical composition? It is also a direction for further research to find out its toxic components and its toxic mechanism and how to reduce toxicity and enhance or maintain the efficacy of Tiekuaizi.

## 6 Predictive analysis of quality markers

### 6.1 Predictive analysis of quality markers based on phylogeny and evidence of secondary metabolite specificity of the original plants

The genus *Chimonanthus* belongs to Calycanthaceae and is characteristic to China, mainly in the mountainous areas of subtropical river valleys south of the Qinling Mountains, west of the Yunnan–Guizhou Plateau, and north of the Nanling Mountains (Dai, 2012). Chinese scholars have divided the genus into eight species and one variety, including *C. nitens, C. praecox*, *C. salicifolius*, *C. grammatus*, *C. baokangensis*, *C. campanulatus*, *C. zhejiangensis*, *C. Grammatus. anhuiensis*, *C. caespitosa*, and *C. campanulatus* var. *guizhouensis* (Li et al., 2021). Among them, *C. nitens* and *C. praecox* are widely distributed species in Guizhou, and their roots, stems, leaves, flower buds, and fruits are used for medicinal purposes (Li and Zou, 2018). The genus *Chimonanthus* has been extensively studied due to its rich production of volatile oils, flavonoids, coumarins, and other bioactive metabolites, and these active substances are now widely used in medicine, pesticides, spices, food, and oil (Li and Zou, 2018; Liu et al., 2018). Coumarins are the main physiologically active substances in the medicinal plants of this genus (Li and Zou, 2018; Liu et al., 2018; Wang et al., 2012; Shu et al., 2019). Through the analysis of the genetic relationship, the difference of components, and the endemic characteristics of the genus of *Chimonanthus* plants, the coumarins in Tiekuaizi can be used as an important basis and feasible way to determine the quality markers.

### 6.2 Predictive analysis of quality markers based on the correlation between the components and efficacy

The theory of properties and actions of Chinese medicinal is a high-level summary of its properties and characteristics. The “four properties and five flavors” is one of the core contents, and “the five flavors” theory is often used as an important basis in clinical medication and combination of medicines and, therefore, is one of the bases for determining quality markers (Yue et al., 2023). Tiekuaizi is pungent, bitter, and warm in nature, entering the lung, liver, heart, and bladder meridians (Guizhou Provincial Drug Administration, 2019), and it is used for the treatment of rheumatic paralysis, bruises, and swelling pains. In the medicinal theory of Chinese medicine, the therapeutic material basis of “bitter” and “pungent” should have their characteristics and functional properties. The bitter flavor can have drying and expelling effects, and it has the effect of clearing away fire and heat, dipping fire and storing yin, and opening up the bowels. In the relationship between bitter principles and meridians, bitter medicines belong to the lung, liver, and stomach meridians. In the relationship between bitter principles and metabolites, those with bitter flavor mostly contain flavonoids, volatile oils, alkaloids, quinones, and glycosides, while bitter–warm and bitter–cold medicines use volatile oils and alkaloids as the main sources of properties and flavors, respectively (Zhang et al., 2016). “Pungent” has the ability to move and disperse and has the effect of dispersing, moving Qi and blood, etc. It mainly enters the heart, spleen, and lung meridians. The therapeutic material includes alkaloids, volatile oils, terpenoids, and glycosides (Zhang M. et al., 2018). In view of this, the coumarins and their glycosides, flavonoids, alkaloids, and volatile oils in Tiekuaizi are the main material basis for the “pungent and bitter” flavor and for the attribution of meridians, and the Q-markers for Tiekuaizi should be selected among the coumarins and their glycosides, alkaloids, and volatile oils.

Coumarins such as isofraxidin, scopoletin, fraxin, and scopolin are the main therapeutic material basis of Tiekuaizi (Luo et al., 2023), which have been found to have anti-inflammatory and analgesic effects, improve cardiovascular and cerebrovascular diseases, improve disorders of glucose and lipid metabolism, and regulate immunity and anti-tumor effects, which have great similarities with traditional efficacy. Tiekuaizi is commonly used as an ethnic medicine for alleviating rheumatic pain and bruises. Therefore, it is concluded that coumarin metabolites represented by scopoletin, etc., should be the main active ingredients to clear heat and remove toxins, resolve swelling, and alleviate pain and can be considered Q-markers of Tiekuaizi.

### 6.3 Predictive analysis of quality markers based on measurability

Based on original plant affinities and the correlation between traditional medicinal properties and metabolites, the Tiekuaizi Q-marker was selected mainly among alkaloids, coumarins, and volatile oils. However, the volatile oils are complex, and the two plants contain different metabolites, making them unsuitable for selection as Q-markers. Alkaloids and coumarins are suitable for determination by chromatographic methods. Researchers have established stable and simple methods for the determination of coumarin-like metabolites in Tiekuaizi, including scopoletin, scopolin, isofraxidin, fraxin, and scoparone (Zhong and Feng, 2017; Bai et al., 2010; Tang et al., 2015; Xiao et al., 2019; Zhou et al., 2013). Calycanthoside, 6, 7, 8-trimethoxycoumarin, and tomenin can also be identified by techniques such as LC–MS chromatographic techniques (Li et al., 2013; Wang, 2020). The above substances are both specific and active, while metabolites that can be easily determined by chromatography are more suitable candidates of Q-markers of Tiekuaizi. In addition, the alkaloid component of Tiekuaizi, calycanthine, has also established a content determination method (Wan et al., 2022). This component shows a good antibacterial effect (Zhang et al., 2009). However, its overdose induces more serious toxic reactions (Knapp et al., 2023), and its toxic-effect relationship needs further study. It is speculated that calycanthine is not only a toxic substance showing slight toxicity but also a pharmacodynamic substance, and it can also be used as a quality marker.

### 6.4 Predictive analysis of quality markers based on blood-accessible chemical components

In a pharmacokinetic study of the main pharmacodynamic components of Tiekuaizi (Zhang J. et al., 2018), the metabolic reactions of scopoletin and scopolin are similar, with different metabolites in different biological samples. The main metabolic reactions in urine and feces are hydrolysis, isomerization, and reduction, and those in plasma are prototype, hydrolysis, reduction, and glucuronidation. A total of three coumarin prototypes (scopoletin, scopolin, and isofraxidin) and 11 metabolites were identified in the study. Through the identification of metabolite products and analysis of their main metabolic pathways, it was revealed that these three coumarin metabolites underwent metabolic reactions such as glucuronidation, sulfonation, and reduction in rats, which are important blood-entry components. These results can provide the pharmacokinetic reference for the selection of Tiekuaizi Q-markers.

## 7 Conclusion

In this paper, based on a review of the current status of research on the chemical composition and pharmacological effects of Miao medicine Tiekuaizi from Guizhou, the screening and identification of Q-markers for Tiekuaizi based on their chemical composition, relevance to clinical use, measurable metabolites, traditional medicinal efficacy, and metabolism of blood components was analyzed and validated in the literature and research data, and coumarins, alkaloid metabolites, were suggested as potential Q-markers for Tiekuaizi, such as scopoletin, scopolin, isofraxidin, fraxin, scoparone, calycanthoside, 6, 7, 8-trimethoxycoumarin, tomenin, and calycanthine. This was done to screen and identify Tiekuaizi Q-markers and establish it as the quality control standard, which can provide a reference for the collection and processing, pharmaceutical production, and effectiveness and safety of clinical applications of Miao medicine Tiekuaizi.

## 8 Discussion

Tiekuaizi has a promising future in anti-inflammatory, immunomodulatory, anti-tumor, hypoglycemic, cardiovascular protection, and anti-viral aspects, but there are still many issues to be resolved in the research on Tiekuaizi. First, the pharmacokinetic and toxicity evaluation of Tiekuaizi metabolites need to be improved. The specific metabolic processes of Tiekuaizi in the body are unclear, and corresponding *in vivo* pharmacokinetic tests should be carried out to elucidate the absorption, transformation, distribution, and excretion processes of Tiekuaizi in the human body and identify the main pharmacodynamic substance basis for their efficacy. Toxicity evaluation and risk avoidance of Tiekuaizi are also key issues that need to be addressed before clinical application. Preliminary toxicity evaluation and investigation of the relationship between the toxicity and efficacy of Tiekuaizi metabolites should be carried out as extensively as possible in future studies, on the basis of which the effective concentration and safe concentration ranges should be further defined, with a view to providing a reference for rational and safe clinical medication. Second, histological techniques should be used to clarify the mechanism of action of Tiekuaizi metabolites. The current means of research on Tiekuaizi is relatively backward compared to the cutting-edge exploration of mechanisms in recent years. Therefore, on the basis of clarifying the action of Tiekuaizi metabolites, relying on genomics, transcriptomics, proteomics, and metabolomics as well as cutting-edge technologies such as single-cell sequencing and epigenetic assays, multi-dimensional detection and screening of more prominent and in-depth action targets of the active metabolites of Tiekuaizi can provide a more thorough and accurate understanding of its pharmacological mechanism of action.
